# Autoantibodies and Sjogren’s Syndrome in Multiple Sclerosis, a Reappraisal

**DOI:** 10.1371/journal.pone.0065385

**Published:** 2013-06-12

**Authors:** Andrew J. Solomon, William Hills, Zunqiu Chen, James Rosenbaum, Dennis Bourdette, Ruth Whitham

**Affiliations:** 1 Department of Neurological Sciences, University of Vermont College of Medicine, Burlington, Vermont, United States of America; 2 Department of Ophthalmology, Oregon Health & Science University, Portland, Oregon, United States of America; 3 Department of Public Health & Preventive Medicine, Oregon Health & Science University, Portland, Oregon, United States of America; 4 Departments of Medicine and Cell Biology, Oregon Health & Science University, Portland, Oregon, United States of America; 5 Department of Neurology, Oregon Health & Science University, Portland, Oregon, United States of America; 6 Portland Veterans Affairs Medical Center, MS Center of Excellence West, Portland, Oregon, United States of America; Innsbruck Medical University, Austria

## Abstract

**Background:**

Rheumatologic diseases may cause neurologic disorders that mimic multiple sclerosis (MS). A panel of serum autoantibodies is often obtained as part of the evaluation of patients suspected of having MS.

**Objectives:**

To determine, in light of recently revised diagnostic criteria for MS, neuromyelitis optica, and Sjogren’s Syndrome, if testing for autoantibodies in patients with a confirmed diagnosis of MS would reveal a frequency or demonstrate a clinical utility divergent from previous reports or lead to identification of undiagnosed cases of Sjogren’s Syndrome.

**Methods:**

Convenience sample cross-sectional study of MS patients recruited from the OHSU Multiple Sclerosis Center.

**Results:**

Autoantibodies were detected in 38% (35/91) of patients with MS and were not significantly associated with disease characteristics or severity. While four patients had SSA antibodies, none met diagnostic criteria for Sjogren’s Syndrome.

**Conclusions:**

Rheumatologic autoantibodies are frequently found in MS patients and are not associated with disease severity or systemic rheumatologic disease. Our demonstration of the low specificity of these autoantibodies suggests that the diagnostic utility and cost-effectiveness of testing is not supported when there is strong clinical suspicion of MS and low clinical suspicion of rheumatologic disease.

## Introduction

Central nervous system manifestations of a number of rheumatologic diseases, particularly Sjogren’s Syndrome, can mimic multiple sclerosis (MS).[1]–[2] As a consequence, serum autoantibodies, such as ANA, SSA, SSB, rheumatoid factor, anticardiolipin antibodies, and lupus anticoagulant, are frequently included in the diagnostic workup of patients suspected of having MS. Limited and conflicting data has been reported in studies investigating the prevalence and clinical significance of these autoantibodies found in patients who have a confirmed diagnosis of MS and have no evidence of an additional coexisting rheumatologic syndrome. [3]–[24] Whether a diagnosis of rheumatologic disease in a patient with MS indicates an increased risk of concurrent autoimmune disease or a misdiagnosis of MS can also be difficult to determine. Sjogren’s Syndrome in particular has been reported to be common among patients diagnosed with MS and there has been suggestion that neurological manifestations of Sjogren’s Syndrome might be mistaken for MS. [23], [25]–[26] Interpretation of previous literature is especially challenging given recent development of improved diagnostic criteria for MS and Sjogren’s Syndrome. [27]–[28] Furthermore, most studies of autoantibodies and Sjogren’s Syndrome in patients with MS were completed prior to the recognition of neuromyelitis optica (NMO) and NMO spectrum disorders (NMOSD) as distinct from MS [29]. The increased prevalence of autoantibodies and related autoimmune diseases, including Sjogren’s Syndrome in particular, among NMO/NMOSD patients [30], suggests the possibility that most previous studies of autoimmune antibodies in MS populations [3]–[4], [6]–[7], [9]–[11], [13]–[24] may have been affected by inclusion of NMO/NMOSD patients.

We sought to reevaluate the frequency of autoantibodies in an MS population with diagnoses confirmed by Revised McDonald Criteria criteria [31] in which NMO/NMOSD had been excluded, assessed whether presence of autoantibodies had any clinical significance and determined whether any patients met current diagnostic criteria [27] for Sjogren’s Syndrome.

## Methods

Patients with MS were recruited from the Multiple Sclerosis Center of Oregon at Oregon Health & Science University (OHSU). Recruitment was limited to female patients due to the infrequent diagnosis of Sjogren’s Syndrome in males. The diagnosis of MS was confirmed using 2005 Revised McDonald Criteria [31]. The 2010 revisions to the McDonald Criteria [28] had not yet been developed at the time of enrollment of the study. Patients with relapsing-remitting MS (RRMS), secondary progressive MS (SPMS), progressive- relapsing (PRMS) and primary progressive MS (PPMS) were included in the study.

Exclusion criteria included known diagnoses of NMO/NMOSD or a known diagnosis of a rheumatologic disease or syndrome that could mimic Sjogren’s Symdrome as outlined revised international classification criteria for Sjogren’s Syndrome [27]. Exclusions included systemic lupus erythematosus, antiphospholipid antibody syndrome, rheumatoid arthritis, hepatitis C infection, lymphoma, graft-versus-host disease, lymphoma, human T-lymphotropic virus Type I infection, human immunodeficiency virus infection and previous head or neck radiation. However, patients currently prescribed medications that might cause dry eye or dry mouth were not excluded.

Recruitment was completed via a convenience sample. From November 2009 through April 2010, successive patients were offered participation in the study during new consultation and follow-up outpatient visits.

Patient demographic and clinical data were collected via chart review. Participants completed a brief survey that included validated questions for ocular and oral symptoms from the revised international classification criteria for Sjogren’s Syndrome. [27] Each participant also completed a self-reported Expanded Disability Status Scale (self-EDSS). [32] This instrument has been demonstrated to correlate with EDSS score as determined by neurological exam. Serum was drawn for antinuclear antibody (ANA), extractable nuclear antigen antibodies (SSA and SSB), rheumatoid factor (RF), anticardiolipin antibodies (aCLs), and lupus anticoagulant (LA).

ANA and RF testing were performed by Kaiser Permanente NW, 13705 NE Airport Way Portland, OR 97230. A sample with a positive ANA with either homogenous or rim pattern was automatically tested for anti-DS DNA antibodies. RF was negative with a value of less than 10 IU/mL. ENA, aCLs, and LA testing was performed at OHSU. For anticardiolipin antibody IgG and IgM, values equal or greater than 20 PL units were considered positive. For anticardiolipin Antibody IgA a value equal or greater than 15 PL units was considered positive. A multi-test panel with a number of phospholipids was used to detect LA. If one of the screening assays was positive, a confirmatory assay was performed (Dilute Viper Venom Confirm or Hexagonal APTT). LA was considered present when one or both confirmatory assays were positive.

Patients with positive SSA or SSB were subsequently offered a Schirmer’s test. This was performed by a neuro-ophthalmologist and included the placement of one drop of 0.5% proparacaine on each eye prior to testing. Patients with a diagnosis of MS who were found to have positive SSA or SSB were also offered testing for serum NMO IgG. This test was performed by Mayo Medical Lab, 200 First St. SW, Rochester, MN 55905.

Analysis of the data was performed using the fisher exact test, two samples t test, and chi square for comparisons.

### Ethics

This study was approved by the OHSU Institutional Review Board (IRB). Informed consent was obtained from each subject in person prior to participation in the study. Participants provided written informed consent, and this process was documented in an IRB-approved consent form. This consent procedure was approved by our IRB research ethics committee.

## Results

### Patient Demographics

Ninety-one female patients with MS participated in the study. Seventy-four had been diagnosed with RRMS, 14 with SPMS, 3 with PPMS, and none with PRMS. Demographic and MS disease characteristics identified via medical record review and self-reported EDSS are presented in Table 1.

**Table 1:** Demographic, Clinical, and Paraclinical Characteristics.

Patients with Multiple Sclerosis	(n:91)
Age (yrs)	48.2±11.9
Years since disease onset	15.6±11.0
Self-EDSS score	4.3±1.6
Spinal cord involvement	42/56 (75%)
CSF-Abnormal OCB or IgG	33/38 (87%)
Last relapse (yrs)	5.30±5.58
New lesion-last MRI	28/72 (39%)
Enhancing lesion-last MRI	3/72 (4%)
Corticosteroids, last ≤3 mos	5/84 (6%)
Current DMT	None: 23/91 (25%)IfnB 1a IM: 26/91 (29%)IfnB 1a SC: 12/91 (13%)IfnB 1b SC: 1/91(1%)
	Glatiramer :21/91 (23%)Natalizumab: 7/91 (8%)Rituximab: 1/91 (1%)

Denominator reflects that information regarding imaging, CSF, and steroid use was not available for all patients. “Spinal cord involvement”: presence of lesions on any previous imaging. “last MRI”: refers to a previous reference scan regardless of interval. IfnB 1a IM: Interferon Beta 1a intramuscular, IfnB 1a SC: Interferon Beta 1a subcutaneous, IfnB 1b SC: Interferon Beta 1b subcutaneous.

### Autoantibodies in MS

Autoantibodies were detected in 35/91 (38%) of MS patients. One autoantibody was detected in 27/35(77%) and two autoantibodies were detected in 8/35(23%). Detailed results of autoantibody testing are presented in Table 2.

**Table 2:** Detection of Autoantibodies in Patients with MS.

	MS
**ANA 1∶40**	14/78 (18%)
**ANA 1∶80**	2/78(3%)
**ANA 1∶160**	6/78 (9%)
**ANA 1∶1280**	1/78 (1%)
**SSA**	4/91 (4%)
**SSB**	0/91
**RF >10**	1/91
**aCL IgG >20**	2/88 (2%)
**aCL IgM >20**	11/88 (13%)
**aCL IgA >15**	1/88 (1%)
**LA**	2/91 (2%)

ANA: antinuclear antibody, SSA and SSB: extractable nuclear antigen antibodies, RF: rheumatoid factor, acL: anticardiolipin antibody, LA: lupus anticoagulant.

* ANA was not drawn in 13/91 patients, and aCLs were not drawn on 3/91 patients.

ANA was positive in 23/78 (31%), including 7/78(10%) with titers equal or greater than 1∶160. Reflex anti-DS DNA was automatically performed in 10 patients and one patient with ANA of 1∶40 tested positive for anti-DS DNA. This patient had no history or exam findings suggestive of rheumatologic disease, imaging was not suggestive of vasculitis, NMO IgG testing completed prior to participation in the study was negative, all additional antibodies tested in the study were negative, and history, exam, and imaging remained consistent with confirmed diagnosis of MS.

SSA was positive in 4/91 (4%) patients. SSB was negative in all patients and RF was mildly elevated at a value of 11 in one patient. Anticardiolipin antibodies(aCLs) were found in 14 patients (16%) and no patient had more than one positive aCL. 11/14 (79%) were low positive (20–30 PL), 3/14 (21%) were moderately positive (31–80 PL) and none were high positive (>80 PL). LA was found in 2/91(2%).

Detection of autoantibodies by MS subtype was also assessed. Statistical comparisons were not made due to small sample size. ANA was positive in 19/74 (26%) patients with RRMS, 3/14 (21%) with SPMS, and 1/3 (33%) with PPMS. Anticardiolipin antibodies were found in 12/74 (16%) of patients with RRMS, 1/14 (7%) with SPMS, and 1/3 (33%) with PPMS. SSA was detected in 1/74 (1%) with RRMS, 3/14 (21%) with SPMS, and 0/3 (0%) with PPMS. Lupus anticoagulant was found in 1/74 (1%) with RRMS, 0/14 (0%) with SPMS, and 1/3 (33%) with PPMS.

### Associations with Autoantibody Detection

In MS patients, the presence of each autoantibody was compared to clinical and laboratory MS disease characteristics. There was no statistically significant relationship between positive ANA, SSA, aCLs, or LA and self-EDSS score, duration from last relapse, new lesion on most recent MRI, the presence of spinal cord disease, current disease modifying therapy, corticosteroid use in the last 3 months, or a previous history of abnormal oligoclonal bands or elevated IgG in CSF. Ocular or oral symptoms were also not significantly associated with positive ANA, SSA, aCLs, or LA.

### Sjogren’s Syndrome Symptoms and Related Testing

All patients completed the revised international classification criteria questions for Sjogren’s Syndrome for ocular and oral symptoms. [27] Thirteen (14%) patients reported having “daily, persistent, troublesome dry eyes for more than 3 months”, 11(12%) reported “a recurrent sensation of sand or gravel in the eyes” and 8(9%) reported “using tear substitutes more than 3 times a day”. Twenty three (25%) patients reported “a daily feeling of dry mouth for more than 3 months”, two patients (2%) reported “recurrently or persistently swollen salivary glands as an adult” and 28(31%) reported they “frequently drink liquids to aid in swallowing dry food”.

Four patients with MS were found to have positive SSA. Per study protocol, all four underwent Schirmer’s test; a result equal to or less than 5 mm in either eye was considered abnormal. Demographic data, MS phenotype, response to ocular and oral symptom survey, Schirmer’s test results, and autoantibody testing results are presented in Table 3. Referral for minor salivary gland biopsy, measurement of salivary flow, sialography, or salivary scintigraphy was not pursued in any patient. A diagnosis of Sjogren’s Syndrome requires either positive SSA/SSB or positive minor salivary gland biopsy, and an additional 3 out of 6 diagnostic criteria. [27] A diagnosis of Sjogren’s Syndrome in our patients was therefore assessed using the results of SSA/SSB positivity, Schirmer’s test, and responses to ocular and oral symptom questions. No patient fulfilled 4 out of the 6 required items for the diagnosis of Sjogren’s Syndrome. Per the study protocol, all 4 MS patients with positive SSA also had their serum tested for NMO IgG, and all four were negative.

**Table 3:** Characteristics of Patients with Positive SSA.

Age/sex	Dx	Schirmer’s (OD/OS)	Ocular sympt	Oral sympt	NMO IgG	ANA	RF	aCLs	LA
46 F	RRMS	5 mm/4 mm	neg	pos	neg	n/a	neg	IgA and Igg: neg, IgM: 28	neg
69 F	SPMS	35 mm/13 mm	neg	neg	neg	1∶80	neg	neg	neg
69 F	SPMS	12 mm/6 mm	neg	pos	neg	1∶40	neg	neg	neg
67 F	SPMS	5 mm/7 mm	neg	neg	neg	Neg	neg	IgA and IgM: neg, IgG: 24	neg

## Discussion

In this study, autoantibodies were frequently detected in patients with a diagnosis of MS. Detection of autoantibodies was not significantly associated with demographic, clinical or paraclinical disease characteristics in patients with MS. Although symptoms and antibodies compatible with Sjogren’s Syndrome were not uncommon in patients with MS, no patient met diagnostic criteria for Sjogren’s Syndrome. This study suggests that screening for rheumatologic autoantibodies in patients where there is strong suspicion of MS and low suspicion of rheumatologic disease is not diagnostically or clinically informative. Our study did not reveal a unique autoantibody profile in patients with MS.

Elevated ANA was particularly common and was found in 31% of patients with MS. More specifically ANA was found in 18% at a dilution of 1∶40, 3% at a dilution of 1∶80, and 9% at a dilution of 1∶160 and 1% at a dilution higher than 1∶160. Although this was similar to the frequency of 23–33% identified by many studies, [3]–[4], [9], [15]–[16], [22] elevated ANA has also been detected in higher (47–81%) [12], [18], [22] and lower (3%–19%) frequency in MS patients [5], [19]–[21], [23] in additional literature. It is important to note that ANA has been detected at a frequency of 32% at 1∶40 dilution, 13% at 1∶80, 5% at 1∶160 and in 3% at 1∶320 in healthy individuals. [33] In agreement with previous studies, detection of RF was rare in our study. [15].

An association between aCLs, myelitis and optic neuritis has been suggested previously [14], raising the possibility that this data was affected by patients with unrecognized NMMO/NMOSD. However, aCLs were detected relatively frequently in MS patients in our study (16%) where patients with NMO/NMOSD were excluded. This was comparable to numerous previous studies [5]–[9], [12], [14]–[15], [17], [22], [24] identifying aCLs in 6–21% of MS patients including two studies where exclusion of NMO/NMOSD patients from analysis was likely [5], [12], both identifying aCLs in 6% of MS patients. ACLs have also been detected at a frequency of 1–9% in numerous studies of healthy populations. [34]–[36].

In our study, SSA was detected in 4% of MS patients and there was no detection of SSB. This finding was similar to previous studies that have identified SSA in 3–13% and SSB in 1–2%. [3], [12], [23] Contrary to previous studies, where Sjogren’s syndrome was identified frequently in an MS population [23], [25]–[26], we did not find any patients with occult Sjogren’s Syndrome. Our findings may reflect our exclusion of NMO/NMOSD patients, differing research methodologies, or the use of more recent standardized diagnostic criteria. [27], [31] Our results are in agreement with a similar previous study reported prior to refinement of MS and Sjogren’s diagnostic criteria. [37] Presuming a relationship between Sjogren’s Syndrome and NMO/NMOSD [30], we tested MS patients with positive SSA for serum NMO IgG. NMO IgG testing was negative in all 4 of these cases. Our study confirms that detection of SSA without a diagnosis of Sjogren’s Syndrome is not uncommon in patients with MS. Furthermore, dry eye and dry mouth were not infrequent in MS patients. While the reasons for this finding are unclear, it’s possible that anticholinergic effects of numerous medications including disease modifying therapies and therapies prescribed for MS-related symptoms might have been the cause.

We detected autoantibodies in 38% of MS patients. While autoantibodies are commonly found in patients with MS, in some studies at a frequency reported to be higher than controls, [3], [9], [12], [15], [19] their significance beyond an epiphenomenon of immune activation remains unclear. Some but not all previous studies have suggested an association between clinical phenotype, frequency of exacerbations or severity of disability, and the detection of autoantibodies. [4], [8], [12], [14], [15] It is possible that potential inclusion of NMO/NMOSD patients may have influenced these results. Our data is in agreement with studies [3]–[4], [6]–[7], [9], [10], [13], [22], [24] that have identified no association with clinical severity. Of note, two recent studies have suggested that MRI measurements of disease burden, including T2 lesion volume, may be associated with the presence of antiphospholipid autoantibodies in patients with MS. [5], [8] Further data is needed to confirm the significance of this finding.

A panel of autoantibodies, including ANA, SSA/SSB, RF, aCLs, and LA is often routinely tested in the workup of a diagnosis of MS. This approach contributes to increased healthcare costs without any benefit. At our institutions it costs approximately $700 to test for the autoantibodies assessed in our study. In addition, detection of these autoantibodies may lead to further unnecessary testing and consultations. Although rheumatologic syndromes may present with CNS manifestations, the frequency of autoantibodies detected in patients with a confirmed diagnosis of MS in this study questions the diagnostic usefulness and cost-effectiveness of this approach when there is not a “red flag” [1] resulting in clinical suspicion of an alternative diagnosis.

There were limitations to this study. There was potential for referral, temporal, and selection bias. Fluctuation of autoantibody levels over time and due to disease modifying therapies [5]–[6], [20] may limit conclusions based on a single serum evaluation. Although there is data supporting our methodology, consensus is lacking for optimal screening methods for Sjogren’s Syndrome in this population. [38] Minor salivary gland biopsy may be the most sensitive method for diagnosing Sjogren’s Syndrome, we did not perform this invasive procedure in this study and it is possible this test may have confirmed a diagnosis in one or more of the patients with positive SSA antibodies. In addition, our study design included only female MS patients given the much lower frequency of Sjogren’s Syndrome in men, and this may have led to a higher frequency of autoantibody detection than would be expected in an MS population including both men and women.

### Conclusions

Autoantibodies associated with rheumatologic disease are frequently identified in patients with MS. However, autoantibody detection (specifically ANA, SSA, SSB, RF, aCLs, and LA) in our study did not provide clinically useful information regarding MS disease characteristics or lead to alternative diagnoses when the clinical suspicion for MS was high and the suspicion for rheumatologic disease was low. Clinicians should reconsider clinical utility and cost-effectiveness of comprehensive autoantibody testing in patients when a diagnosis of MS is strongly suspected.

## References

[1] Miller DH, Weinshenker BG, Filippi M, Banwell BL, Cohen JA, et al (2008) Differential diagnosis of suspected multiple sclerosis: a consensus approach. Mult Scler 14: 1157–74. 18805839 10.1177/1352458508096878 PMC2850590

[2] Delalande S, de Seze J, Fauchais AL, Hachulla E, Stojkovic T, et al (2004) Neurologic manifestations in primary Sjögren syndrome: a study of 82 patients. Medicine 83: 280–91. 15342972 10.1097/01.md.0000141099.53742.16

[3] de Andrés C, Guillem A, Rodríguez-Mahou M, López Longo FJ (2001) Frequency and significance of anti-Ro (SS-A) antibodies in multiple sclerosis patients. Acta Neurol Scand 104: 83–7. 11493223 10.1034/j.1600-0404.2001.104002083.x

[4] Collard RC, Koehler RP, Mattson DH (1997) Frequency and significance of antinuclear antibodies in multiple sclerosis. Neurology 49: 857–61. 9305354 10.1212/wnl.49.3.857

[5] Garg N, Zivadinov R, Ramanathan M, Vasiliu I, Locke J, et al (2007) Clinical and MRI correlates of autoreactive antibodies in multiple sclerosis patients. J Neuroimmunol 187: 159–65. 17512610 10.1016/j.jneuroim.2007.04.008

[6] Heinzlef O, Weill B, Johanet C, Sazdovitch V, Caillat-Zucman S, et al (2002) Anticardiolipin antibodies in patients with multiple sclerosis do not represent a subgroup of patients according to clinical, familial, and biological characteristics. J Neurol Neurosurg Psychiatry 72: 647–9. 11971055 10.1136/jnnp.72.5.647 PMC1737868

[7] Roussel V, Yi F, Jauberteau MO, Couderq C, Lacombe C, et al (2000) Prevalence and clinical significance of anti-phospholipid antibodies in multiple sclerosis: a study of 89 patients. J Autoimmun 14: 259–65. 10756088 10.1006/jaut.2000.0367

[8] Stosic M, Ambrus J, Garg N, Weinstock-Guttman B, Ramanathan M, et al (2010) MRI characteristics of patients with antiphospholipid syndrome and multiple sclerosis. J Neurol. 257: 63–71. 10.1007/s00415-009-5264-6 19633967

[9] Tourbah A, Clapin A, Gout O, Fontaine B, Liblau R, et al (1998) Systemic autoimmune features and multiple sclerosis: a 5-year follow-up study. Arch Neurol 55: 517–21. 9561980 10.1001/archneur.55.4.517

[10] Vilisaar J, Wilson M, Niepel G, Blumhardt LD, Constantinescu CS (2005) A comparative audit of anticardiolipin antibodies in oligoclonal band negative and positive multiple sclerosis. Mult Scler 11: 378–80. 16042217 10.1191/1352458505ms1208oa

[11] Chapman J (2004) The interface of multiple sclerosis and antiphospholipid antibodies. Thromb Res 114: 477–81. 15507281 10.1016/j.thromres.2004.06.016

[12] Szmyrka-Kaczmarek M, Pokryszko-Dragan A, Pawlik B, Gruszka E, Korman L, et al (2012) Antinuclear and antiphospholipid antibodies in patients with multiple sclerosis. Lupus 21: 412–20. 22074845 10.1177/0961203311427550

[13] Sastre-Garriga J, Reverter JC, Font J, Tintoré M, Espinosa G, et al (2001) Anticardiolipin antibodies are not a useful screening tool in a nonselected large group of patients with multiple sclerosis. Ann Neurol 49: 408–11. 11261519

[14] Karussis D, Leker RR, Ashkenazi A, Abramsky O (1998) A subgroup of multiple sclerosis patients with anticardiolipin antibodies and unusual clinical manifestations: do they represent a new nosological entity? Ann Neurol 44: 629–34. 9778261 10.1002/ana.410440408

[15] Spadaro M, Amendolea MA, Mazzuconi MG, Fantozzi R, Di Lello R, et al (1999) Autoimmunity in multiple sclerosis: study of a wide spectrum of autoantibodies. Mult Scler 5: 121–5. 10335521 10.1177/135245859900500209

[16] Barned S, Goodman AD, Mattson DH (1995) Frequency of anti-nuclear antibodies in multiple sclerosis. Neurology 45: 384–5. 7854544 10.1212/wnl.45.2.384

[17] D’Olhaberriague L, Levine SR, Salowich-Palm L, Tanne D, Sawaya KL, et al (1998) Specificity, isotype, and titer distribution of anticardiolipin antibodies in CNS diseases. Neurology 51: 1376–80. 9818863 10.1212/wnl.51.5.1376

[18] Dore-Duffy P, Donaldson JO, Rothman BL, Zurier RB (1982) Antinuclear antibodies in multiple sclerosis. Arch Neurol 39: 504–506. 7049132 10.1001/archneur.1982.00510200046008

[19] De Keyser J (1998) Autoimmunity in multiple sclerosis. Neurology 38: 371–374. 10.1212/wnl.38.3.371 3347339

[20] Verdun E, Isoardo G, Oggero A, Ferrero B, Ghezzi A, et al (2002) in multiple sclerosis patients before and during IFN-beta 1b treatment: are they correlated with the occurrence of autoimmune diseases? J Interferon Cytokine Res 22: 245–255. 11911808 10.1089/107999002753536220

[21] Seyfert S, Klapps P, Meisel C, Fischer T, Junghan U (1990) Multiple sclerosis and other immunologic diseases. Acta Neurol Scan 81: 37–42. 10.1111/j.1600-0404.1990.tb00928.x 2330813

[22] Ciccarelli O, Bagnato F, Mainero C, Salvetti M, Paolillo A, et al (2000) Antinuclear antibodies and response to IFN-b therapy in relapsing-remitting multiple sclerosis. Mult Scler 6: 137–139. 10871823 10.1177/135245850000600301

[23] de Seze J, Devos D, Castelnovo G, Labauge P, Dubucquoi S, et al (2001) The prevalence of Sjögren syndrome in patients with primary progressive multiple sclerosis. Neurology 57: 1359–63. 11673571 10.1212/wnl.57.8.1359

[24] Liedorp M, Sanchez E, van Hoogstraten IM, von Blomberg BM, Barkhof F, et al (2007) No evidence of misdiagnosis in patients with multiple sclerosis and repeated positive anticardiolipin antibody testing based on magnetic resonance imaging and long term follow-up. J Neurol Neurosurg 78: 1146–8. 10.1136/jnnp.2007.117713 PMC2117534 17878195

[25] Pericot I, Brieva L, Tintoré M, Río J, Sastre-Garriga J, et al (2003) Myelopathy in seronegative Sjögren syndrome and/or primary progressive multiple sclerosis. Mult Scler 9: 256–9. 12814172 10.1191/1352458503ms905oa

[26] Sandberg-Wollheim M, Axéll T, Hansen BU, Henricsson V, Ingesson E, et al (1992) Primary Sjogren’s syndrome in patients with multiple sclerosis. Neurology 42: 845–7. 1565240 10.1212/wnl.42.4.845

[27] Vitali C, Bombardieri S, Jonsson R, Moutsopoulos HM, Alexander EL, et al (2002) European Study Group on Classification Criteria for Sjogren’s Syndrome. Classification criteria for Sjogren’s syndrome: a revised version of the European criteria proposed by the American-European Consensus Group. Ann Rheum Dis 61: 554–8. 12006334 10.1136/ard.61.6.554 PMC1754137

[28] Polman CH, Reingold SC, Banwell B, Clanet M, Cohen JA, et al (2011) Diagnostic criteria for multiple sclerosis: 2010 revisions to the McDonald criteria. Ann Neurol 69: 292–302. 21387374 10.1002/ana.22366 PMC3084507

[29] Wingerchuk DM, Lennon VA, Pittock SJ, Lucchinetti CF, Weinshenker BG (2006) Revised diagnostic criteria for neuromyelitis optica. Neurology 66: 1485–9. 16717206 10.1212/01.wnl.0000216139.44259.74

[30] Wingerchuk DM, Weinshenker BG (2012) The emerging relationship between neuromyelitis optica and systemic rheumatologic autoimmune disease. Mult Scler 18: 5–10. 10.1177/1352458511431077 22146604

[31] Polman CH, Reingold SC, Edan G, Filippi M, Hartung HP, et al (2005) Diagnostic criteria for multiple sclerosis: 2005 revisions to the "McDonald Criteria". Ann Neurol 58: 840–6. 16283615 10.1002/ana.20703

[32] Bowen J, Gibbons L, Gianas A, Kraft GH (2001) Self-administered Expanded Disability Status Scale with functional system scores correlates well with a physician-administered test. Mult Scler 7: 201–6. 11475445 10.1177/135245850100700311

[33] Tan EM, Feltkamp TE, Smolen JS, Butcher B, Dawkins R, et al (1997) Range of antinuclear antibodies in "healthy" individuals. Arthritis Rheum 40: 1601–11. 9324014 10.1002/art.1780400909

[34] Shi W, Krilis SA, Chong BH, Gordon S, Chesterman CN (1990) Prevalence of lupus anticoagulant and anticardiolipin antibodies in a healthy population. Aust N Z J Med20: 231–6. 10.1111/j.1445-5994.1990.tb01025.x 2115326

[35] Vila P, Hernández MC, López-Fernández MF, Batlle J (1994) Prevalence, follow-up and clinical significance of the anticardiolipin antibodies in normal subjects. Thromb Haemost 72: 209–13. 7831653

[36] Petri M (2000) Epidemiology of the antiphospholipid antibody syndrome. J Autoimmun 15: 145–51. 10968901 10.1006/jaut.2000.0409

[37] Noseworthy JH, Bass BH, Vandervoort MK, Ebers GC, Rice GP, et al (1989) The prevalence of primary Sjogren’s syndrome in a multiple sclerosis population. Ann Neurol 25: 95–8. 2913935 10.1002/ana.410250117

[38] Sánchez-Guerrero J, Pérez-Dosal MR, Celis-Aguilar E, Cárdenas-Velázquez F, Soto-Rojas AE, et al (2006) Validity of screening tests for Sjogren’s syndrome in ambulatory patients with chronic diseases. J Rheumatol 33: 907–11. 16541477

